# Rapid DNA and RNA isolation from few or single cells using low-cost NAxtra magnetic nanoparticles

**DOI:** 10.1038/s41598-025-05770-y

**Published:** 2025-07-02

**Authors:** Eirin Johannessen Starheim, Adeel Manaf, Adnan Hashim, Niklas Nonboe Andersen, Erlend Ravlo, Wei Wang, Vidar Langseth Saasen, Nina-Beate Liabakk, Sten Even Erlandsen, Per Arne Aas, Lars Hagen, Magnar Bjørås

**Affiliations:** 1 Department of Clinical and Molecular Medicine (IKOM), Norwegian University of Science and Technology (NTNU), 7491 Trondheim, Norway; 2 Lybe Scientific, Erling Skjalgssons Gate 1, 7030 Trondheim, Norway; 3 Department of Microbiology, Oslo University Hospital and University of Oslo, 0372 Oslo, Norway; 4 Centre of Embryology (CRESCO), University of Oslo, 0313 Oslo, Norway; 5 Proteomics and Modomics Experimental Core Facility (PROMEC) at NTNU, 7491 Trondheim, Norway

**Keywords:** Biological techniques, Biotechnology, Molecular biology

## Abstract

A novel, cost-effective nucleic acid (NA) isolation method for purifying total NA, DNA, or RNA from both two- and three-dimensional cell cultures has been developed at the Norwegian University of Science and Technology utilizing NAxtra magnetic nanoparticles. This method achieves comparable yields to existing isolation kits while offering significant improvements in cost and processing speed. However, the original protocol was not optimized for small-cell numbers or single-cell applications. Given the growing interest in single-cell and rare-cell population studies, there is a critical need for more sensitive isolation techniques. In this study, we have enhanced the sensitivity of the NAxtra-based isolation method to facilitate mid- to high-throughput purification from as few as 10,000 cells down to single cells. Automated processing using KingFisher systems enables the rapid handling of 96 samples within 12–18 min. Our findings indicate that this method not only matches but can exceed the performance of existing alternatives in (RT)-qPCR detection while being significantly more economical and efficient. Additionally, it enables the extraction of high-quality RNA suitable for transcriptomics analyses from limited cell quantities, including single cells. This advancement holds substantial promise for improving the accessibility and efficiency of NA research, particularly in studies involving scarce cellular materials.

**Supplementary Information:**

The online version contains supplementary material available at 10.1038/s41598-025-05770-y.

## Introduction

In molecular research, there are instances where isolation of RNA or DNA from very low cell numbers is desirable, particularly when few cells are available for extraction, e.g., rare cells, or when characteristics of single cells are of interest. This pertains to numerous research areas, including developmental biology, immunology, neuroscience and oncology. Certain immune cells, stem cells, and circulating tumor cells (CTCs) are examples of rare cell populations that are actively studied for their roles in immune response mechanisms, tissue regeneration, and cancer progression^1–4^. Additionally, CTCs isolated from the blood of cancer patients can be sequenced to identify mutation spectrums representative of the tumor heterogeneity^5,6^. This information is not only useful for research purposes but also has potential in a clinical setting. For instance, certain actionable mutations are predictive of a patient’s response to treatment with specific drugs^7^. Examples of more common cell types that may be studied in minimal quantities, are neuronal or glial cell populations in cerebral subregions^8^. Mice are frequently employed as mammalian model organisms for this purpose, where the number of each cell type available for analysis in each region can often be fewer than 10,000 cells^8–10^. Single-cell transcriptomics and (epi)genomics can provide information that is lost with bulk methods, thereby improving our understanding of various biological processes. This includes novel insights into cell classification, differentiation and development, and the molecular mechanisms driving disease progression, such as those underlying clonal evolution in cancer^11,12^. However, obtaining information from a few or single cells is challenging, as it involves the extraction of trace amounts of RNA or DNA. Consequently, specialized methods are required.

There are two typical approaches for DNA or RNA extraction from a few or single cells: nucleic acid (NA) purification and NA release by cellular lysis in a buffer compatible with downstream reactions. One example of the former approach is a modified version of the conventional phenol–chloroform method of NA extraction, which has been used to isolate RNA from single cells^13^. However, these samples may be contaminated with residual phenol or chloroform, which are hazardous chemicals that can impact downstream reactions, thereby skewing the data^14^. To avoid this, one may use phenol/chloroform-free commercial kits capable of single-cell RNA purification, such as the AllPrep DNA/RNA Micro Kit (QIAGEN), AllPrep DNA/mRNA Nano Kit (QIAGEN, mRNA purification only), Quick-DNA/RNA Microprep Plus Kit (Zymo Research), PicoPure RNA Isolation Kit (Applied Biosystems), Single Cell RNA Purification Kit (Norgen Biotek), or GenElute Single Cell RNA Purification Kit (Sigma-Aldrich). Regarding DNA purification, the AllPrep DNA/RNA Micro Kit (QIAGEN) can be used for inputs as low as ten cells. At the same time, the AllPrep DNA/mRNA Nano Kit (QIAGEN) and Quick-DNA/RNA Microprep Plus Kit (Zymo Research) can extract DNA down to the single-cell level. However, current commercial purification methods for single cells are expensive, and all except the bead-based AllPrep DNA/mRNA Nano Kit rely on spin columns, resulting in low-throughput procedures with an estimated duration of roughly 3–5 h to produce 96 eluates (Supplementary table 6).

The PicoPure DNA Extraction Kit (Applied Biosystems) and various kits for single-cell real-time qPCR (e.g., CellsDirect kit, Single Cell-to-C_T_ kit), single-cell whole genome/transcriptome amplification (e.g., RepliG, Ampli1^15^) and protocols for single-cell RNA sequencing (e.g., SMART-Seq(2/3)^16–18^, FLASH-seq^19^) utilize the second approach of NA extraction where NA are released by cell lysis in a buffer compatible with subsequent reactions. In addition, high-throughput platforms such as the Fluidigm C1 (HT-IFC) and ICELL8 (Takara Bio) can be employed to process up to 800 or 1800 single cells, respectively. Although downstream assays can be performed on these unpurified samples, the reaction efficiency may be reduced due to the presence of inhibiting cell and buffer components^20^. For sequencing, this may result in increased noise if a larger number of pre-amplification cycles becomes necessary^21^. Additionally, the high cost of the commercial kits restricts the number of samples that can be processed on a limited budget.

Recently, we developed a low-cost NA isolation procedure for mammalian cells and organoids at the Norwegian University of Science and Technology (NTNU)^22^. The procedure is based on NAxtra (Lybe Scientific), a bead-based NA isolation technology created in response to a national shortage of commercial NA isolation kits in Norway during the COVID-19 pandemic^23^. With this technology, purification is achieved by cell lysis in a customized buffer followed by magnetic extraction of released NA through their adsorption to superparamagnetic, silica-coated, iron oxide nanoparticles (Supplementary figure 1). Following several wash steps, the purified NA are eluted in a suitable buffer or nuclease-free water. Herein, we showcase a high-sensitivity version of this procedure (Supplementary figure 1), allowing simple, rapid, inexpensive purification of total NA, RNA, or DNA down to the single-cell level. When automated on the robot systems KingFisher Duo Prime Magnetic Particle Processor (Thermo Scientific) or KingFisher Flex Purification System (Thermo Scientific), simultaneous processing of up to 96 samples can be performed automatically in 12 min for total NA isolation, and 18 min (+ nuclease incubation time) for RNA/DNA purification. The extracted NA are compatible with downstream applications, including (RT-)qPCR and next-generation sequencing (NGS).

## Results

### Nucleic acid extraction from ultra-low cell inputs

Current commercial purification methods for small cell inputs, including single cells, face challenges such as limited throughput and high costs, highlighting the need for developing techniques that enhance accessibility and efficiency. The NAxtra-based method presented here allows fast, low-cost extraction of RNA and/or DNA from 96 samples simultaneously for cell inputs down to single cells without the need for carrier RNA, proteinase K, or beta-mercaptoethanol. Initially, the cells are lysed using a custom buffer, facilitating the binding of NA to magnetic nanoparticles (Supplementary figure 1). After separating the NA from contaminants, total RNA or DNA is obtained through nuclease treatment. The NA are then purified through a series of wash steps, during which the magnetic beads remain magnetized, and are subsequently eluted. The most similar kit currently on the market is the bead-based AllPrep DNA/mRNA Nano kit (QIAGEN). Therefore, this kit was used as a benchmark for the novel NAxtra-based procedure (see Supplementary table 1 for a comparison of key features).

**Fig. 1:**
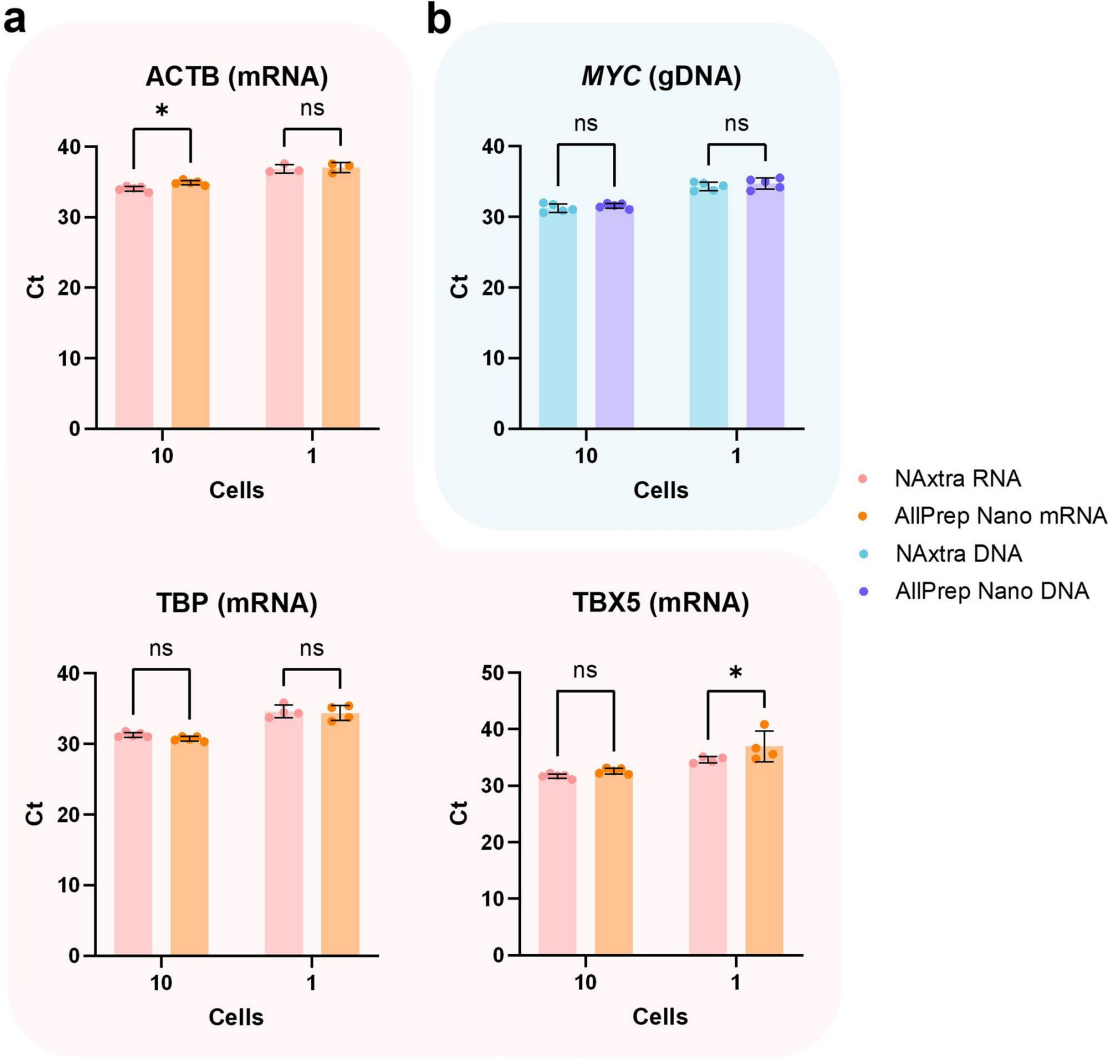
Nucleic acid extraction from ultra-low cell inputs (1–10 cells of HAP1), comparing the NAxtra-based method to the AllPrep DNA/mRNA Nano kit (QIAGEN). Average cycle threshold (Ct) values (± 1 SD) for (**a**) mRNA targets (ACTB, TBP, TBX5) and (**b**) Genomic DNA (gDNA) target (*MYC*) amplified by (RT-)qPCR. Independent replicate extractions (n = 5) are shown as dots. Statistical analysis was performed by two-way ANOVA with Šídák’s multiple comparisons test, in which ns (non-significant) = adjusted *P* > 0.05 and * = adjusted *P* ≤ 0.05.

Extraction of DNA and RNA was performed for 1 and 10 sorted cells of an adherent human cell line (HAP1) (Fig. 1). This cell line contains a near-haploid genome, requiring that genomic DNA (gDNA) isolation from individual cells achieve single-allele resolution to be effective. The extraction yield was assessed by (RT-)qPCR of a gDNA target (*MYC*) and three mRNA targets (Actin beta, ACTB; TATA-Box Binding Protein, TBP; T-Box Transcription Factor 5, TBX5). *MYC* is a proto-oncogene that is frequently studied in cancer research, also at the single-cell level^24,25^, making it a clinically relevant target for gDNA isolation. ACTB mRNA is expressed from a housekeeping gene that exhibits high and relatively stable expression within distinct cell types^26,27^, making it a suitable target for evaluating efficiency of RNA isolation for single cells. In addition, other mRNA targets were included to further showcase the sensitivity of the RNA isolation. Specifically, *TBP* can be considered a housekeeping gene and reportedly displays significantly lower expression than *ACTB*^28–30^. Moreover, *TBX5* is involved in forelimb and heart development^31^ and is expected to exhibit very low expression in HAP1 cells as they do not derive from those tissues. NAxtra and AllPrep RNA samples were eluted in equal volumes. For the most part, the RT-qPCR results show comparable mRNA detection between the two methods. However, NAxtra samples exhibit superior ACTB mRNA detection for 10 cells, with an average Ct difference of 0.83 (± 0.21), and superior detection of TBX5 mRNA for single cells, with an average Ct difference of 2.39 (± 1.24). This indicates that the corresponding AllPrep RNA samples may only contain roughly 50–65% or 10–45% of the respective target mRNA compared to NAxtra RNA samples. Furthermore, NAxtra purifies total RNA^22^, while the AllPrep kit purifies only mRNA. Therefore, the favorable method for RNA isolation largely depends on the desired RNA species, in addition to cost and speed. For DNA samples, the elution volume corresponded to the minimal requirement for each method, namely 25 µl for AllPrep Nano and 5 µl for NAxtra. The relatively large elution volumes for the AllPrep DNA samples can give rise to stochastic issues for downstream reactions, including qPCR and NGS. Such effects are not observed for the detection of *MYC* gDNA by qPCR, where *MYC* is detected in all single-cell samples for both methods. This may be explained by an increase in *MYC* gene copy number in the HAP1 cell line, which is a common occurrence in cancer cells^32–34^. Although the NAxtra method allows a 5-fold reduction in elution volume for DNA, this does not result in proportional improvement in DNA detection. Therefore, the AllPrep kit may be superior in terms of DNA isolation efficiency. Still, the two methods exhibit similar performance for qPCR with equal volume input and are expected to follow that trend for other downstream applications with restrictive input volumes, such as library preparation for NGS. In addition, the cost of materials and reagents is reduced ~ 16-fold when using NAxtra (Supplementary table 4). Moreover, the manual NAxtra method can be performed for 96 samples in a similar or shorter time frame as required for the isolation of 24 samples when using the AllPrep kit. In sum, both methods have their advantages and limitations, and the favorable choice depends on the number of samples, the research budget, time constraints, the NA species of interest, and NA yield/volume requirements for downstream applications.

### Cell input range for manual and automated NA extraction

**Fig. 2:**
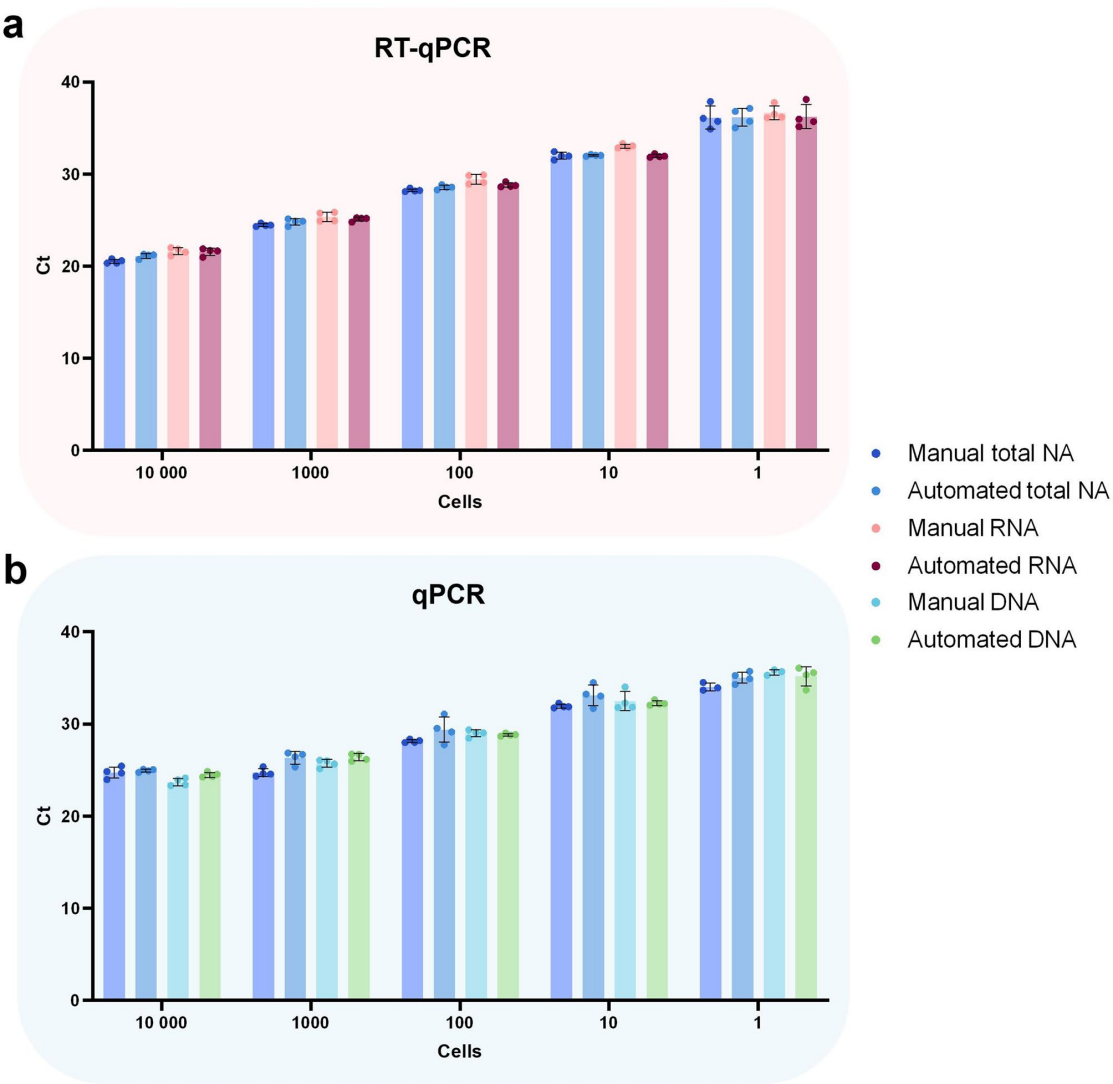
Manual and automated (KingFisher Flex Purification System, Thermo Scientific) extraction of total nucleic acid (NA), RNA, or DNA from 1 to 10,000 cells (HAP1) using the NAxtra-based method. Average cycle threshold (Ct) values (± 1 SD) for (**a**) mRNA target (ACTB) and (**b**) gDNA target (*MYC*) amplified by (RT-)qPCR. Independent replicate extractions (n = 4) are shown as dots.

Automation saves time, reduces variation and is particularly useful when large numbers of samples are to be processed. Commercial purification kits for single cells, such as the AllPrep DNA/mRNA Nano kit, do not currently offer protocols for automated extraction. The NAxtra method reported here can be automated on any magnetic particle processor designed for NA purification applications. For instance, we have implemented it on the robot systems KingFisher Duo Prime Magnetic Particle Processor (Thermo Scientific) and KingFisher Flex Purification System (Thermo Scientific) for up to 12 or 96 samples, respectively. To illustrate the cell input range of the NAxtra method, total NA, DNA, and RNA were extracted manually and automatically from 1 to 10,000 cells of an adherent human cell line (HAP1). Extraction yield was assessed by (RT-)qPCR of a genomic DNA target (*MYC*) and an mRNA target (ACTB) (Fig. 2). The extraction efficiency is shown to be consistent for cell inputs up to 10,000 cells, with the relationship between Ct and cell number (log scale) being linear for the detection of ACTB (R^2^ ≥ 0.999 for total NA and R^2^ ≥ 0.998 for RNA) and *MYC* (R^2^ ≥ 0.941 for total NA and R^2^ ≥ 0.990 for DNA) in NAxtra samples. The automated NAxtra protocol exhibits similar performance to the manual NAxtra method and provides a tenfold improvement in processing speed relative to the manual AllPrep DNA/mRNA Nano kit (Supplementary table 5), making it a favorable procedure for high-throughput NA isolation from few or single cells.

### Extraction of nucleic acids from ultra-low cell inputs of various cell types

**Fig. 3:**
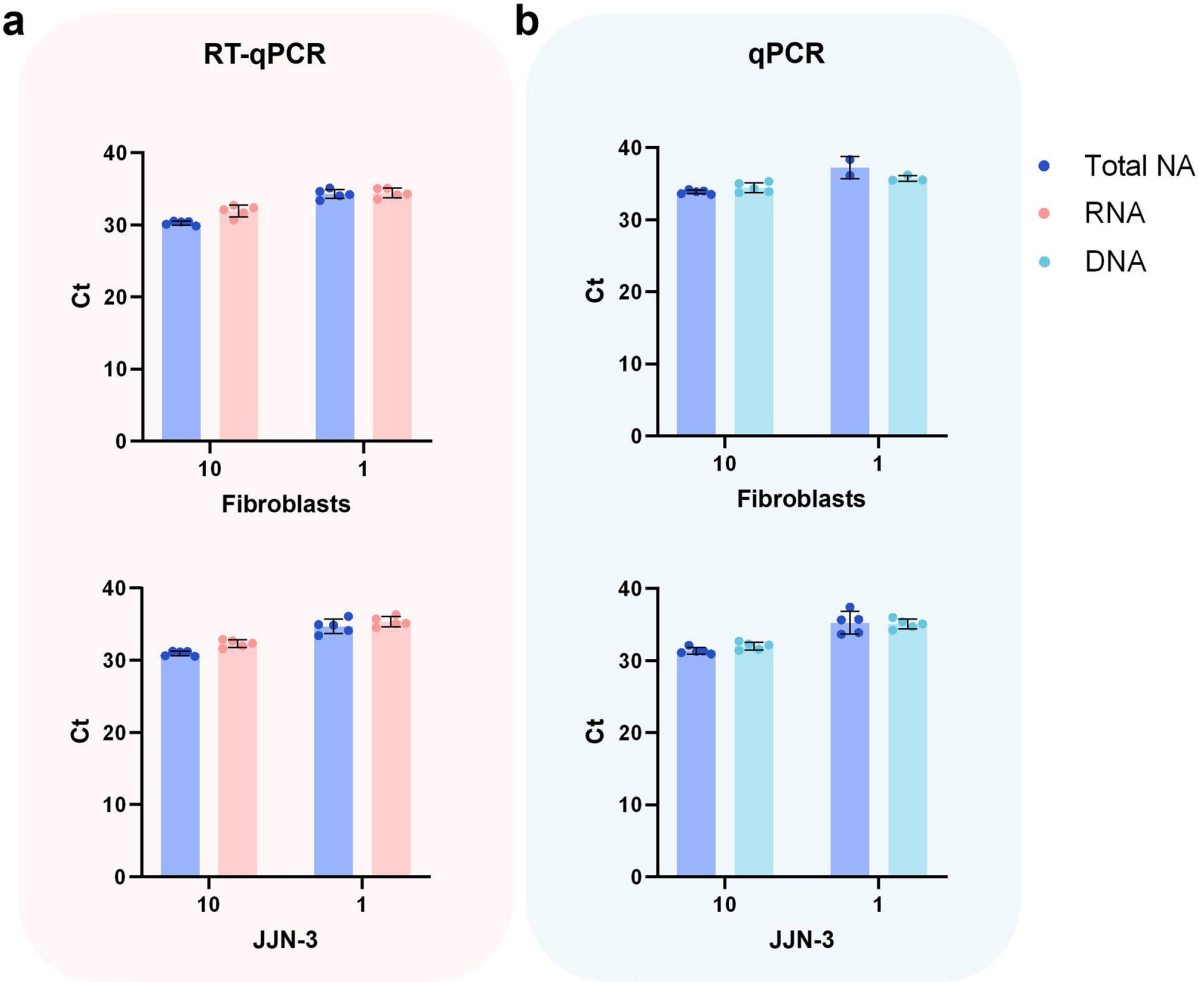
Manual extraction of total nucleic acid (NA), RNA, or DNA from 1–10 cells of adherent primary cells (human fibroblasts) and a suspension cell line (JJN-3, human multiple myeloma) using the NAxtra-based method. Average cycle threshold (Ct) values (± 1 SD) for (**a**) mRNA target (ACTB) and (**b**) DNA target (*MYC*) amplified by (RT-)qPCR. Independent replicate extractions (n = 5) are shown as dots.

NA detection for single cells can vary between cell types due to differences in gene copy number or gene expression level. For instance, the SNARE technique published by Strotman and colleagues in 2013 enabled the detection of gDNA (*GADPH*) from single-cell samples (50%, n = 6) but for only one of the three tested cell lines^35^. In Figs. 1 and 2, we show that the NAxtra-based method provides high single-cell sensitivity for the adherent cell line HAP1. In addition, the NAxtra method has successfully been used to extract high-quality RNA from single peripheral blood mononuclear cells (PBMCs)^36^. To further assess the compatibility of the NAxtra method with other cell types, extraction of total NA, RNA, and DNA was performed for ultra-low inputs (1 and 10 cells) of a suspension cell line (JJN-3) and human primary fibroblasts (Fig. 3). JJN-3 is a well-established model for multiple myeloma and can be considered representative of bone marrow-derived plasma cells^37^. Fibroblasts are widely studied in numerous research areas where single-cell analyses have been instrumental, including wound healing and tissue repair, immunology, cancer biology and fibrosis^38–40^. The extraction yield was evaluated by (RT-)qPCR cycle threshold (Ct) values for a gDNA target (*MYC*) and an mRNA target (ACTB) (Fig. 3). The results show single-cell sensitivity for gDNA and mRNA for both cell types when using NAxtra. In sum, the NAxtra method is compatible with various cell types, including suspension and adherent cell lines, as well as primary cells.

### Single-cell transcriptomics

**Fig. 4:**
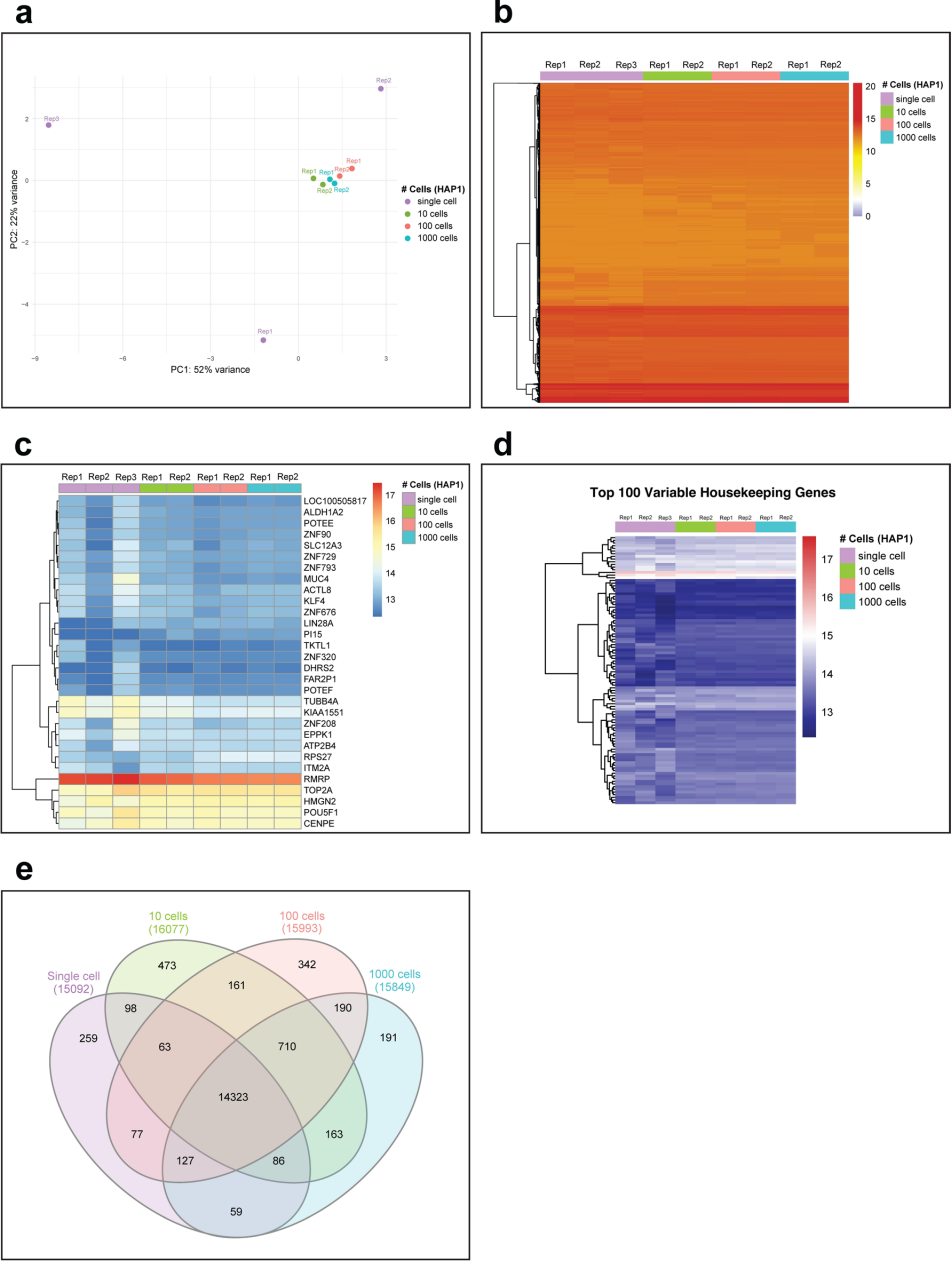
Transcriptome analysis of NAxtra-based RNA-Seq data from 1000 cells to single-cell level. (**a**) Principal Component Analysis (PCA) of RNA-Seq data showing sample clustering across different cell numbers (1000, 100, 10, and single cells). While single-cell samples show slightly more variation (as expected), all samples maintain consistent clustering patterns, demonstrating the robustness of the protocol across different cell numbers. (**b**) Global gene expression heatmap across all samples and conditions. The hierarchical clustering dendrogram and intensity scale (0–20) represent normalized gene expression values. The color gradient from blue to red indicates low to high expression levels, showing consistent expression patterns across different cell numbers. (**c**) Heatmap of top 30 expressed genes across samples. Expression values are scaled (13–17), with darker blue indicating lower expression and red indicating higher expression. Gene clustering reveals similar expression patterns maintained across different cell numbers. (**d**) Expression profile of top 100 variable housekeeping genes (Expression values are scaled 13–17). The consistent expression patterns of housekeeping genes across different cell numbers validate the down-scaled protocol’s ability to maintain reliable gene expression measurements even at the single-cell level. (**e**) Venn diagram illustrating the overlap in number of genes discovered across different cell numbers.

Transcriptome analyses of few or single cells can provide insights into cellular heterogeneity and rare cell types. To assess the compatibility of the NAxtra-based isolation method with downstream transcriptome analyses using NGS, total RNA was extracted from 1, 10, 100, and 1000 cells of an adherent human cell line (HAP1). Dual-indexed libraries were generated with the SMART-Seq Stranded Kit (Takara) and sequenced on NovaSeq X Plus with 50 bp paired-end reads. The NAxtra-based RNA sequencing (RNA-Seq) data showed consistent gene expression patterns across the different cell numbers, with a slight expected increase in variation for single cells (Fig. 4). Gene clustering analysis of the top 100 variable housekeeping genes and the 30 most expressed genes further confirmed the consistent detection of the expression patterns down to single-cell. These data sets demonstrate the robustness of NAxtra technology in providing reliable and efficient RNA isolation from ultra-low cell inputs, compatible with NGS for transcriptome analyses down to the single-cell level.

**Fig. 5:**
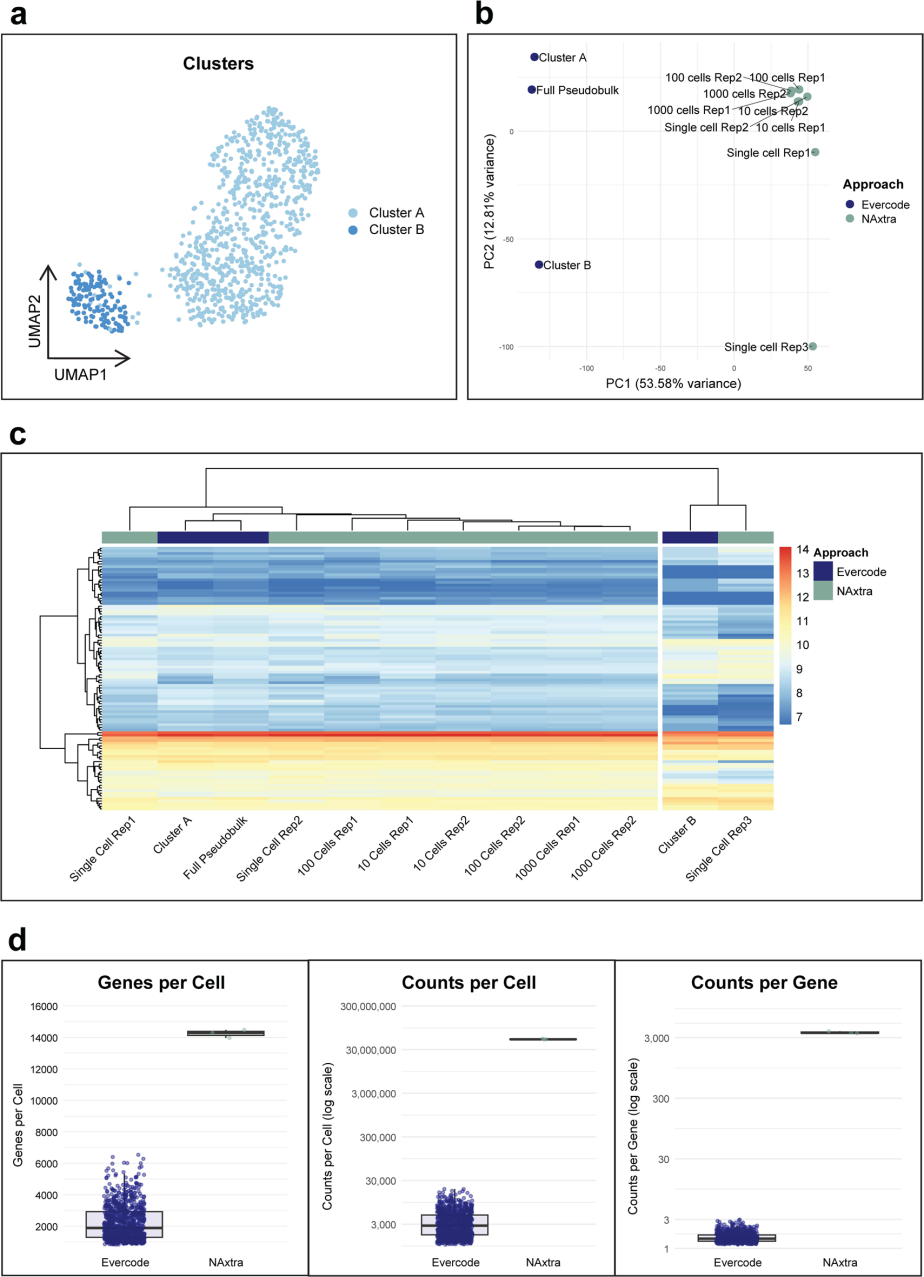
Transcriptome analysis of RNA-Seq data from 1000, 100, 10, and single cells using the NAxtra/SMART-Seq-based approach, and 886 single cells using the Evercode WT v2 approach. (**a**) Cell clustering for the Evercode data, distinguishing two clusters in the full pseudobulk, here called Cluster A (750 cells) and Cluster B (136 cells). (**b**) Principal Component Analysis (PCA) of NAxtra/SMART-Seq and Evercode RNA-Seq data showing clustering across the samples, with expected variation between the two methods and similarities in clustering of samples within each method. (**c**) Heatmap of the top 100 variable genes for PC2 (Expression values are scaled 7–14). Sample clustering reveals similar expression patterns between Evercode Cluster B and NAxtra Single Cell Rep3 as well as between the remaining Evercode and NAxtra samples. (**d**) Median genes per cell, counts per cell and counts per gene for the single cells of the NAxtra-based and Evercode approach.

To compare the NAxtra-based approach to a commonly used single-cell NGS approach, the state-of-the-art Evercode technology (Parse Biosciences) with combinatorial barcoding was used. Evercode is similar to the NAxtra/SMART-Seq well-based approach in providing uniform transcript coverage of the whole transcriptome^41^. For the Evercode approach, RNA-seq data was generated for 886 single cells of the same HAP1 cell population as utilized for NAxtra/SMART-Seq. Cell clustering for the Evercode data distinguished two clusters in the full pseudobulk, hereafter called Cluster A (750 cells) and Cluster B (136 cells) (Fig. 5a), indicating the presence of two HAP1 subpopulations. When comparing the two methods by Principal Component Analysis (PCA) (Fig. 5b), the observed variation is in line with previous comparisons of combinatorial barcoding approaches to well-based approaches^41^. Upon further examination, the main variation between samples within each of the two approaches, represented by the PC2 loadings, was comparable across the two methods. This is illustrated by the similar gene expression patterns in the gene clustering analysis of the top 100 genes contributing to this principal component (Fig. 5c). In fact, the Evercode Cluster B and the NAxtra Single Cell Rep 3 clustered together, and the remaining samples clustered together, signifying that two HAP1 subpopulations were detected in both approaches. This is consistent with the variation observed at the single-cell level for the NAxtra-based approach in Fig. 4. The median number of genes per cell was 14,290 for the single-cell samples of the NAxtra-based approach and 1888 for the Evercode approach (Fig. 5d), with the respective average number of reads per cell being ~ 86,000,000 and ~ 83,000 (Supplementary table 7). Due to the difference in number of reads per cell, the gap in detected genes is expected. Additionally, it coincides with an anticipated saturation in genes detected per cell for the NAxtra-based approach when exceeding 80 million reads per cell. Meanwhile, the median number of counts per cell and counts per gene did not exhibit saturation. In fact, these values were ~ 18,500-fold and ~ 2500-fold higher for the NAxtra approach, respectively (Fig. 5d, Supplementary table 7). After accounting for the ~ 1000-fold increase in number of reads per cell relative to Evercode, these differences remain substantial. Altogether, these data demonstrate that compared to the high-throughput Evercode approach, which captures a broad overview of cellular heterogeneity, the high-resolution gene expression profiles obtained using the NAxtra-based approach can enable a more detailed characterization of individual cells.

## Discussion

Single-cell qPCR and next-generation sequencing analysis are fundamental to understanding the heterogeneity in disease and healthy tissue. Recent developments in single-cell sequencing have tremendously improved our understanding of cellular pathways. However, the cost of performing these experiments often makes them inaccessible to the majority of researchers. Moreover, most of these kits require a large number of cells to start with and are mostly designed for obtaining single-cell sequencing data. In this study, we demonstrated that the NAxtra-based method enables the isolation of RNA and/or DNA from 10,000 cells down to the single-cell level for both qPCR and NGS, with compatibility across various cell types, i.e., adherent, suspension, and primary cells. Thus, the method is applicable across diverse research areas, including oncology, neuroscience, immunology and developmental biology, where the study of rare cell populations, precious samples, or single cells is crucial for advancing current knowledge. Compared to a benchmark kit from QIAGEN, the manual NAxtra method exhibits comparable (RT-)qPCR performance while offering a ~ 16-fold reduction in cost and substantial improvement in sample processing speed. Importantly, when automated, NAxtra achieved a ~ 10-fold decrease in processing time relative to the benchmark kit. The significant reduction in cost enhances accessibility for a broader range of researchers, particularly those with limited funding. Moreover, the large reduction in processing time, especially when automated, facilitates more rapid data generation, thereby accelerating research timelines. However, the necessary equipment for automation may currently be prohibitively expensive for some laboratories. The gene expression patterns obtained with whole transcriptome RNA-seq of NAxtra samples while down-scaling from 1000 to single cells were consistent, and the observed variation between single cells was in line with expression patterns observed between cells for a state-of-the-art alternative technology, namely Evercode (Parse Biosciences). This provided additional evidence for the reliability and efficiency of the method. In sum, the NAxtra method consistently and effectively isolates NA from ultra-low cell inputs, compatible with downstream applications such as (RT-)qPCR and single-cell transcriptomics, and thus represent a low-cost, rapid alternative to other purification methods.

## Methods

### Cell culture and sorting

Human Cell line HAP1 (Horizon Discovery), JJN-3, and primary fibroblasts, provided by the Department of Clinical and Molecular Medicine (IKOM) at NTNU, were cultured as described previously^22^. At 70–80% confluency, the cells were collected (300 g, 4 °C, 5 min), resuspended in ice-cold PBS, strained through a 35 µm mesh strainer cap, and sorted on a BD FACSAria IIu cell sorter.

### Nucleic acid isolation

#### NAxtra manual extraction

Desired cell numbers were sorted with single-cell precision into a 60 µl (RNA-optimized protocol) or 30 µl (DNA-optimized protocol) mixture containing one-part PBS and two-parts NAxtra LYSIS BUFFER (Lybe Scientific) in a 96-well twin.tec® LoBind® PCR Plate (Eppendorf). An 80 µl (RNA-optimized protocol) or 40 µl (DNA-optimized protocol) mixture of 4 µl NAxtra MAGNETIC BEADS (Lybe Scientific) in isopropanol was added to each sample. Beads were kept in suspension by shaking for 1.5 min at 900 rpm, then centrifuged briefly and collected to the side of the tubes for 1 min using the DynaMag-96 Side Magnet (Invitrogen). The supernatant was removed. For samples to be nuclease-treated, the beads were washed on the magnet with 150 µl absolute ethanol, dried for 2 min, and resuspended in 15 µl (RNA protocol) or 5 µl (DNA protocol) nuclease-free water. DNase treatment of RNA samples was performed for 10 min with 0.4 µl (1–100 cells) or 1 µl (1000–10,000 cells) RNase-Free DNase (QIAGEN), 2 µl Buffer RDD (QIAGEN) and nuclease-free water to a final volume of 20 µl. RNase treatment of DNA samples was performed for 2 min with 8 µg PureLink RNase A (Invitrogen) and nuclease-free water to a final volume of 10 µl. Nuclease-treated samples were combined with 40 µl (RNA protocol) or 20 µl (DNA protocol) NAxtra LYSIS BUFFER (Lybe Scientific), followed by 80 µl (RNA protocol) or 40 µl (DNA protocol) isopropanol. To rebind the treated NA, the beads were kept in suspension by shaking for 1.5 min at 900 rpm, briefly centrifuged, magnetized to the side of the tubes for 1 min, and the supernatant was removed. All NA samples were washed on the magnet, once with 150 µl isopropanol and twice with 150 µl 80% ethanol. The beads were dried for 2 min before resuspension in nuclease-free water or TET buffer for DNA in Fig. 1. Following shaking for 1 min at 900 rpm, eluates were spun down, and the beads were magnetized to the side of the tubes for 1 min to allow transfer of the eluates into a new plate. Elution volumes were 5 µl for DNA in Fig. 1 and for DNA/total NA assessed by qPCR for Fig. 3, while 10 µl was used for all samples in Fig. 2, RNA in Fig. 1, and RNA/total NA assessed by RT-qPCR for Fig. 3.

#### NAxtra on KingFisher Flex

The procedure followed the manual extraction method described earlier, with the specific modifications outlined below. Mixing, washing, and elution steps were automated on the KingFisher Flex Purification System with a 96 Deep-Well Head (Thermo Scientific) and performed in Nunc 96-Well Polypropylene Sample Processing & Storage Microplates (Thermo Scientific). Slight adjustments were made for samples to be nuclease-treated, with resuspension in 20 µl nuclease-free water, DNase treatment of RNA samples with 2.5 µl Buffer RDD (QIAGEN), and final nuclease treatment volumes of 25 µl. Nuclease-treated samples were combined with 30 µl NAxtra LYSIS BUFFER (Lybe Scientific), followed by 90 µl isopropanol. Final elution volumes were 12 µl.

#### AllPrep DNA/mRNA Nano Kit

The AllPrep DNA/mRNA Nano Kit (QIAGEN) is a manual bead-based method for isolation of DNA and mRNA from low-input samples. DNA and mRNA extraction was performed for 1 and 10 sorted cells according to the manufacturer’s instructions, using the minimal required elution volumes of 25 µl for the DNA samples and 10 µl for the mRNA samples.

### Real-time quantitative PCR

(RT-)qPCR was conducted by thermal cycling on a CFX96 Touch Real-Time PCR Detection System (Bio-Rad) with reaction volumes of 10 µl, including triplicate no template controls (NTCs) for each master mix and triplicate no cell controls (NCCs) for each extraction method.

#### DNA amplification (qPCR)

Reactions for amplification of MYC gDNA contained 4.5 µl eluate, 1X MYC TaqMAN Copy Number Assay Hs00834648_cn (Applied Biosystems), and 1X RealQ Plus 2 × Master Mix for Probe (Ampliqon), and were performed with reaction conditions specified in master mix instructions, with an annealing temperature of 62 °C.

#### RNA amplification (RT-qPCR)

Reactions for reverse transcription and amplification of ACTB mRNA contained 4.75 µl eluate, 1X ACTB PrimeTime XL qPCR Assay Hs.PT.39a.22214847 (Integrated DNA Technologies) and 1X qScript XLT 1-Step RT-qPCR ToughMix (Quantabio), and were conducted with reaction conditions specified by the master mix manufacturer, with an annealing temperature of 64 °C.

Reactions for reverse transcription and amplification of TBX5 or TBP mRNA contained 4.3 µl eluate, 100 nM of each target-specific primer (Supplementary table 2), 1X Luna WarmStart® RT Enzyme Mix, 1X Luna® Universal One-Step RT-qPCR Kit (NEB), and were conducted with reaction conditions specified by the reaction mix manual, with an annealing temperature of 60 °C.

### RNA sequencing

#### NAxtra experimental procedure

Total RNA was extracted from replicates of 1, 10, 100, and 1000 sorted cells of the HAP1 cell line using the manual NAxtra method described earlier, with elution in 8 µl nuclease-free water. Dual index libraries were prepared using the SMART-Seq Stranded Kit (Takara) according to protocol guidelines. The libraries were sequenced with 50 bp paired-end reads on NovaSeq X Plus, yielding on average ~ 76 M paired-end reads per sample and ~ 86.1 M paired-end reads per cell for the single-cell replicates (Supplementary table 7). Illumina sequencing was performed by the Norwegian Sequencing Centre, Department of Medical Genetics, Oslo University Hospital, Ullevål.

#### Evercode experimental procedure

Evercode by Parse Biosciences is considered state-of-the-art for whole transcriptome single-cell RNA sequencing with uniform transcript coverage^41^. Evercode Cell Fixation v2 (Parse Biosciences) was conducted for 100,000 sorted HAP1 cells according to manufacturer’s instructions, resulting in ~ 7000 fixed cells. Library preparation with the Evercode Whole Transcriptome v2 Kit (Parse Biosciences) was executed according to kit specifications. Single-cell sequencing was performed with 151 bp paired-end reads on NovaSeq 6000 S4, yielding on average ~ 83 k paired-end reads per cell (Supplementary table 7). Illumina sequencing was conducted by the Norwegian Sequencing Centre, Department of Medical Genetics, Oslo University Hospital, Ullevål.

#### NAxtra data processing and analysis

Raw sequencing data quality was assessed using FastQC (v0.11.9)^42,43^. Adapter trimming and quality filtering were performed using Trim Galore (v0.6.7)^44^. Paired-end reads were processed simultaneously, and poor quality bases and adapter sequences were removed from both reads. FastQC was run on the trimmed reads to verify the improvement in sequence quality. The trimmed reads were aligned to the reference genome using HISAT2 (v2.2.1)^45^. The resulting SAM files were converted to BAM format, sorted by genomic coordinates, and indexed using SAMtools (v1.16.1)^46^. Gene-level quantification was performed using the featureCounts function from the Subread package (v2.0.6)^47^. The resulting count matrix was then used for differential expression analysis using DESeq2 (v1.44.0)^48^ Bioconductor package in R to compare gene expression levels between different experimental conditions.

#### Evercode data processing and analysis

The Split-pipe pipeline (version 1.3.1, Parse Biosciences) was utilized for the preprocessing of the single-cell RNA sequencing data. The resulting count matrix was filtered for empty droplets and to include only the features used during alignment of the NAxtra-based RNA sequencing data. Analysis of the count matrix was performed utilizing the Seurat package (version 5.2.1)^49^. Cells were further filtered based on the distribution of counts per cell, unique genes per cell, and the percentage of mitochondrial genes expressed per cell. The filtered raw counts contained 886 cells. These counts were normalized using log-normalization, and variable features were found using the Seurat ‘vst’ selection method. Data was scaled according to the default Seurat workflow. PCA was run and 15 PCs were chosen for finding neighbours, clustering and UMAP visualization.

#### Combined analysis of NAxtra and Evercode approaches

Single cells from the HAP1 cell line formed two distinct clusters in the Evercode data. These two clusters, along with the combined population, were converted to pseudobulk using the Seurat2PB function from edgeR (version 4.2.2)^50^. These pseudo-counts were combined with the counts from the NAxtra-based RNA sequencing data. Normalization of the combined count-matrix was performed using the variance stabilizing transformation from DESeq2 (version 1.44.0)^48^. A PCA was conducted to assay sample differences. A heatmap was plotted of the features making up the top 100 loadings of PC2, as this PC resembled the difference between samples within approaches. The heatmap was created using pheatmap (version 1.0.12)^51^. The median genes per cell, counts per cell, and counts per gene were calculated for the three NAxtra single-cell samples and for each cell (886) in the Evercode dataset.

### Statistical analyses

Statistical analyses were performed in GraphPad Prism (version 10.4.1) by two-way ANOVA with Šídák’s multiple comparisons test using a significance threshold of 0.05. Results are provided in Supplementary Table 3.

## Electronic supplementary material

Below is the link to the electronic supplementary material.

Supplementary Material 1

## Data Availability

The authors confirm that the data supporting the findings of this study are available within the article and its supplementary materials. The RNA-Seq data has been deposited for public access in the NIH database Gene Expression Omnibus (GEO) (https://www.ncbi.nlm.nih.gov/geo/). The accession codes are GEO Dataset: GSE284534 (NAxtra/SMART-seq) and GEO Dataset: GSE296244 (Evercode). The tokens of the datasets, accessible on https://www.ncbi.nlm.nih.gov/geo/query/acc.cgi?acc=GSE284534 and https://www.ncbi.nlm.nih.gov/geo/query/acc.cgi?acc=GSE296244, are inyfokkkxhsnhah and udopkmgmxncbpsf, respectively.
