# Supplementary material for: Rapid DNA and RNA isolation from few or single cells using low-cost NAxtra magnetic nanoparticles
Source: Sci Rep. 2025 Jul 2;15:22472. doi: 10.1038/s41598-025-05770-y (PMC12214658; doi:10.1038/s41598-025-05770-y)
Supplement: Supplementary file 1 — Supplementary Material 1 [file 41598_2025_5770_MOESM1_ESM.docx]

**Rapid DNA and RNA isolation from few or single cells using low-cost NAxtra magnetic nanoparticles**

*Eirin Johannessen Starheim^1^, Adeel Manaf^1,3,4^, Adnan Hashim^1,3,4^, Niklas Nonboe Andersen^1^, Erlend Ravlo^1^, Wei Wang^1^, Vidar Langseth Saasen^1^, Nina-Beate Liabakk^1^, Sten Even Erlandsen^2^, Per Arne Aas^1^, Lars Hagen,^1,5^, Magnar Bjørås^1,3,4^**

^1^ Norwegian University of Science and Technology (NTNU), Department of Clinical and Molecular Medicine (IKOM), 7491, Trondheim, Norway.

^2^ Lybe Scientific, Erling Skjalgssons gate 1, 7030 Trondheim, Norway.

^3^ Oslo University Hospital and University of Oslo, Department of Microbiology, 0372 Oslo, Norway.

^4^ Centre of Embryology (CRESCO), University of Oslo, 0313 Oslo, Norway.

^5^ Proteomics and Modomics Experimental Core Facility (PROMEC) at NTNU, 7491, Trondheim, Norway.

*E-mail: magnar.bjoras@ntnu.no

**Supplementary table 1**. Key features of the novel NAxtra-based single-cell/small-bulk nucleic acid (NA) isolation procedure compared to the previously developed NAxtra large bulk NA isolation method and the AllPrep DNA/mRNA Nano kit (QIAGEN).

| **Features** | **NAxtra bulk (previous)** | **NAxtra single cell (novel)** | **AllPrep Nano** |
| --- | --- | --- | --- |
| **Cell input** | 100-1,000,000 | 1-10,000 | 1-10,000 |
| **NA output** | Total NA, DNA or RNA | Total NA, DNA or RNA | DNA and mRNA |
| **Format** | 96 deep-well plate or 1.5 ml tubes | 0.2 ml 96 well plate or 0.2 ml tubes | 1.5 ml tubes |
| **Automated version available** | Yes | Yes | No |
| **Magnetic bead input** | Medium/high (20/60 µl) | Low (4 µl) | Medium (20/30 µl) |
| **Binding volume** | High (700 µl) | Low (70/140 µl) | Low/high (220 µl/1.02 ml) |
| **Wash Volume** | High (400 µl) | Low (150 µl) | Low/high (100/500 µl) |
| **Magnetic bead position during washing** | Resuspended | Magnetized | Resuspended |
| **Elution volume** | ≥ 20 µl | ≥ 5 µl | ≥ 10/25 µl |


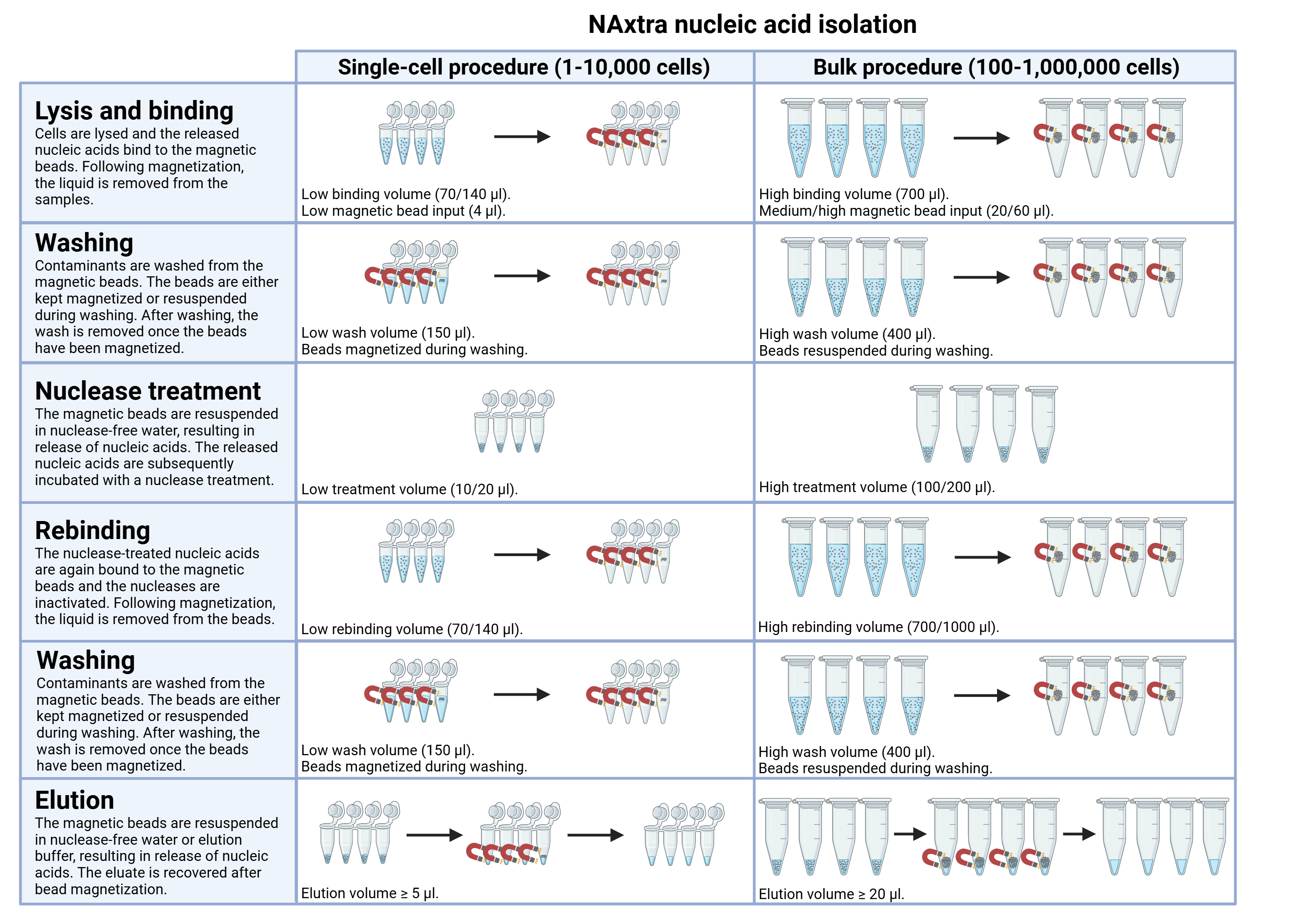
 **Supplementary figure 1**. Schematic of manual NAxtra-based nucleic acid isolation, showing the novel single-cell/small-bulk nucleic acid isolation procedure and the previously developed large-bulk isolation method. The general steps for isolation of RNA or DNA are depicted. For isolation of total NA, the steps for nuclease treatment, rebinding and the second round of washing would be omitted. Created in BioRender. Starheim, E. (2025) https://BioRender.com/c6ei5q1.

**Supplementary table 2**. Primers for amplification of mRNA targets using RT-qPCR.

| **mRNA target** | **Amplicon size (bp)** | **Primer sequence (5’→ 3’)** |
| --- | --- | --- |
| TBP | 121 | Forward: CGGCTGTTTAACTTCGCTTC |
|  |  | Reverse: CCAGCACACTCTTCTCAGCA |
| TBX5 | 115 | Forward: AGGGCATGGAGGGAATCAAA |
|  |  | Reverse: TAACTGGGAAACATCCGCCT |

**Supplementary table 3**. Statistical analysis of mRNA target (ACTB, TBP, TBX5) detection in RNA samples, and gDNA target (MYC) detection in DNA samples, extracted from 1 or 10 sorted HAP1 cells with five independent replicates using NAxtra versus the AllPrep DNA/mRNA Nano kit (QIAGEN).

| **NAxtra vs. AllPrep Nano** | **Statistical significance** | **Adjusted P value** |
| --- | --- | --- |
| Detection of ACTB mRNA from 10 cells | * | 0.0268 |
| Detection of ACTB mRNA from 1 cell | ns | 0.9046 |
| Detection of TBP mRNA from 10 cells | ns | 0.4036 |
| Detection of TBP mRNA from 1 cell | ns | 0.8844 |
| Detection of TBX5 mRNA from 10 cells | ns | 0.5152 |
| Detection of TBX5 mRNA from 1 cell | * | 0.0454 |
| Detection of *MYC* gDNA from 10 cells | ns | 0.6514 |
| Detection of *MYC* gDNA from 1 cell | ns | 0.4846 |

The data was analysed in GraphPad Prism (version 10.4.1) by two-way ANOVA with Šídák’s multiple comparisons test.
Ns (non-significant) = adjusted P > 0.05, * = adjusted P ≤ 0.05.

**Supplementary table 4**. Estimated cost (USD) to obtain 96 sample eluates from low cell inputs through manual nucleic acid isolation using the NAxtra-based method (Lybe Scientific) versus AllPrep DNA/mRNA Nano kit (QIAGEN).

| **Method** | **Component (Supplier, Catalog number)** | **Price^A^ (USD for 96 eluates)** |
| --- | --- | --- |
| **NAxtra** | NAxtra Cells total nucleic acid extraction kit 96 reactions (Lybe Scientific, LSNXC0096) | 172.80^B^ |
|  | Eppendorf twin.tec® LoBind® PCR Plates (VWR, 737-0185) | 6.66 |
|  | RNase-Free DNase Set (QIAGEN, 79254) | 26.88^C^ |
|  | PureLink™ RNase A 20 mg/mL (ThermoFisher Scientific, 12091021) | 0.62 |
|  | Total | **193.20^D^** |
| **AllPrep Nano** | AllPrep DNA/mRNA Nano (QIAGEN, 80272) | 3116.00 |
|  | Total | **3161.00** |

A = Prices obtained from suppliers December 2024. B = Price calculated based on number of preps listed for the kit (96), but reagents should be sufficient for at least twice as many preps due to the small volumes of magnetic beads and lysis buffer utilized for the modified, high-sensitivity NAxtra method. This would reduce the total price of reagents and materials for 96 eluates to 106.8 USD for the NAxtra method, i.e., representing a ~29-fold reduction in price compared to AllPrep Nano. C = Price calculated based on 1 µl DNase input per sample, although cell inputs ≤ 100 cells require only 40% of this volume. D = Price calculated for 50/50 RNA/DNA eluates to be comparable to AllPrep Nano and demonstrates a ~16-fold reduction in price for the NAxtra method.

**Supplementary table 5**. Estimated processing time (h) to obtain 96 sample eluates from low cell inputs through bead-based nucleic acid (NA) isolation using the NAxtra-based method (Lybe Scientific) versus AllPrep DNA/mRNA Nano kit (QIAGEN).

| **Bead-based method** | **Type** | **NA** | **Duration (h for 96 eluates)** |
| --- | --- | --- | --- |
| **NAxtra** | Manual | Total NA | 1 |
|  |  | RNA | 2 |
|  |  | DNA | 2 |
|  | Automated | Total NA | 0.2 |
|  |  | RNA | 0.5 |
|  |  | DNA | 0.4 |
| **AllPrep DNA/mRNA Nano** | Manual | 50/50 mRNA/DNA | 5^A^ |

A = Duration is based on 4 repetitions of the 75 min processing time reported by QIAGEN for 24 eluates, but longer durations may be experienced. The corresponding duration for the NAxtra method would be 2 h if manual and 0.5 h if automated, which represents 2.5-fold and 10-fold reductions in processing time, respectively, compared to AllPrep Nano.

**Supplementary table 6**. Estimated processing time (h) to obtain 96 sample eluates from low cell inputs through nucleic acid (NA) isolation using manual, column-based extraction kits.

| **Column-based method** | **NA** | **Estimated duration (h for 96 eluates)** |
| --- | --- | --- |
| **AllPrep DNA/RNA Micro Kit (QIAGEN)** | 50/50 DNA/RNA | 4.7^A^ |
| **Quick-DNA/RNA Microprep Plus Kit (Zymo Research)** | 50/50 DNA/RNA | 2.7^B^ |
| **PicoPure RNA Isolation Kit (Applied Biosystems)** | Total RNA | 2.7^C^ |
| **Single Cell RNA Purification Kit (Norgen Biotek)** | Total RNA | 2.7^C^ |
| **GenElute Single Cell RNA Purification Kit (Sigma-Aldrich)** | Total RNA | 2.7^C^ |

A = Duration is based on the 35 min processing time reported by QIAGEN for two eluates, assuming a quadrupling in time for 48 eluates, and 2 repetitions. B = Duration is based on the 20 min processing time reported by Zymo Research for one eluate when using Quick-DNA Microprep Plus Kit, assuming a quadrupling in time for 48 eluates, and 2 repetitions. The duration may be extended when using the combined Quick-DNA/RNA Microprep Plus Kit. C = Duration is based on the 20 min purification time for 10 eluates reported by corresponding suppliers, assuming a doubling in time for 24 eluates and 4 repetitions.

**Supplementary table 7**. Key features of the two approaches for whole transcriptome (WT) RNA sequencing, namely NAxtra/SMART-Seq and Evercode WT v2.

| **Features** | **NAxtra/SMART-Seq** | **Evercode** |
| --- | --- | --- |
| **Samples** | 3 single cells, and duplicates of 10, 100, and 1000 cells | 886 single cells |
| **Sample preparation method** | NAxtra single-cell/small-bulk RNA isolation procedure | Evercode Cell Fixation (Parse Biosciences) |
| **Library preparation kit** | SMART-Seq Stranded Kit (Takara) | Evercode WT v2 kit (Parse Biosciences) |
| **Library preparation type** | Well-based | Combinatorial barcoding |
| **Sequencing throughput** | Low-medium | High |
| **Transcript coverage** | Uniform | Uniform |
| **Instrument** | NovaSeq X Plus | NovaSeq 6000 |
| **Flow cell** | 1.5B | S4 |
| **Read format** | Paired end | Paired end |
| **Read length** | 50 bp | 151 bp |
| **Mean reads per sample** | ~ 76,000,000* | ~ 74,000,000 |
| **Mean reads per cell** | ~ 86,000,000** | ~ 83,000 |
| **Median genes per cell** | 14,290 | 1888 |
| **Median counts per cell** | 51,581,544 | 2778 |
| **Median counts per gene** | 3610 | 1.45 |

* = The mean for the 9 samples. ** = The mean for the 3 single-cell samples.
